# Retrospective analysis of bleeding risk with tirofiban in patients with acute ischemic stroke and diabetes mellitus

**DOI:** 10.1097/MD.0000000000049681

**Published:** 2026-07-31

**Authors:** Dongmei Rao, Zhi Li, Biao Guo

**Affiliations:** a Department of Neurology, Yingshang County People’s Hospital, Fuyang City, Anhui Province, China.

**Keywords:** acute ischemic stroke, bleeding risk, blood glucose control, diabetes mellitus, extracranial bleeding, tirofiban

## Abstract

This study aimed to explore the risk of overall extracranial bleeding events with tirofiban in patients with acute ischemic stroke (AIS) and diabetes mellitus and analyze the impact of blood glucose control on this risk. A single-center retrospective cohort study design was used, including 100 patients with AIS and diabetes mellitus admitted between April 2023 and November 2025. Propensity score matching was used to divide patients into the tirofiban group (50 cases) and the control group (50 cases). The primary outcome was the incidence of overall extracranial bleeding events within 7 days after medication administration. Secondary outcomes included types of extracranial bleeding (e.g., gingival bleeding), thrombocytopenia, and neurological function outcomes. Multivariable logistic regression and subgroup analysis were conducted to assess independent risk factors. The incidence of overall extracranial bleeding events in the tirofiban group was 32.0%, significantly higher than the 16.0% in the control group (risk ratio = 2.00, 95% confidence interval = 1.11–3.60, *P* = .021). Gingival bleeding (10.0% vs 2.0%, *P* = .047) and thrombocytopenia (10.0% vs 2.0%, *P* = .047) occurred more frequently in the tirofiban group. Multivariable analysis showed that tirofiban treatment (adjusted odds ratio = 2.45, *P* = .018) and admission random blood glucose (adjusted odds ratio = 1.22, *P* = .032) were independent risk factors for overall extracranial bleeding events. Subgroup analysis revealed that in patients with hemoglobin A1c ≥7.5%, the bleeding risk associated with tirofiban was higher (odds ratio = 3.33, 95% confidence interval = 1.05–10.56, *P*-interaction = .04). In patients with AIS and diabetes mellitus, tirofiban treatment did not significantly increase the risk of intracranial hemorrhage, but it significantly raised the risk of extracranial bleeding events (especially gingival bleeding) and thrombocytopenia. This risk was more pronounced in patients with poor blood glucose control. Comprehensive monitoring of bleeding signs and enhanced glucose management are required in clinical practice.

## 1. Introduction

Acute ischemic stroke (AIS) is one of the leading causes of disability and mortality worldwide.^[1]^ The primary goal of acute-phase treatment in AIS is to restore blood flow and perfusion. In addition to standard intravenous thrombolysis and endovascular therapy, antiplatelet treatment plays a significant role in preventing early stroke recurrence and improving microcirculation in the ischemic region.^[2]^ Tirofiban, a highly selective, short-acting nonpeptide glycoprotein IIb/IIIa receptor antagonist, can rapidly and effectively inhibit the final common pathway of platelet aggregation.^[3]^ In recent years, its application value in AIS treatment has attracted considerable attention, especially in scenarios such as perforating artery infarction and thrombosis prevention during endovascular therapy, where it has shown potential efficacy and relatively acceptable safety.^[4–6]^

However, potent antiplatelet therapy is inevitably associated with bleeding risks. In AIS patients, symptomatic intracranial hemorrhage (sICH) is one of the most severe complications during the acute phase. It is worth noting that diabetes mellitus is a common and significant comorbidity in AIS patients. These patients have a unique pathophysiological condition: prolonged hyperglycemia leads to severe endothelial dysfunction, enhanced platelet reactivity, fibrinolytic system dysregulation, and increased blood–brain barrier permeability.^[7,8]^ These changes collectively create a vulnerable intravascular environment and a “bleeding-prone” pathological foundation. Theoretically, adding potent antiplatelet drugs like tirofiban to this fragile foundation may significantly amplify the overall bleeding risk.

Currently, although some studies have evaluated the safety of tirofiban in the general AIS population,^[9,10]^ there is a lack of research specifically focused on the bleeding risk in the large and high-risk subgroup of AIS patients with diabetes. Existing evidence mainly focuses on intracranial hemorrhage, with insufficient attention to extracranial bleeding events, including gingival bleeding and subcutaneous hematomas. Clinicians facing such patients lack sufficient evidence to comprehensively assess the bleeding risk of tirofiban, particularly regarding all types of bleeding events.

Therefore, it is crucial to clarify the overall and various types of bleeding risks associated with tirofiban in AIS patients with diabetes and identify high-risk subgroups (such as those with poor blood glucose control). This will guide clinicians in making individualized and precise antiplatelet treatment decisions. This study aims to systematically explore the impact of adding tirofiban to routine treatment on the incidence of overall bleeding events (with a special focus on extracranial bleeding) in AIS patients with diabetes, using a single-center retrospective cohort study. Propensity score matching (PSM) will be used to control for confounding biases, and further analysis will investigate the modifying effects of blood glucose control and other factors on bleeding risk.

## 2. Methods

### 2.1. Study design

This study was approved by the Ethics Committee of Yingshang County People’s Hospital. This study is a single-center, retrospective cohort study. We conducted a retrospective analysis of medical records of patients with AIS and diabetes mellitus admitted to the stroke unit and neurology department of our hospital between April 2023 and November 2025. The aim was to investigate whether the addition of tirofiban to routine treatment increased the short-term (within 7 days after medication) bleeding-related risk.

### 2.2. Inclusion and exclusion criteria

Inclusion criteria: age ≥18 years; clinical diagnosis of AIS, confirmed by imaging; a clear diagnosis of type 2 diabetes mellitus or admission fasting/random blood glucose levels meeting the diagnostic criteria; the time window from admission to thrombolysis or endovascular therapy is in accordance with relevant guidelines; complete clinical data, including at least: admission National Institutes of Health Stroke Scale (NIHSS), blood glucose levels, coagulation parameters, blood pressure control, imaging data, and bleeding outcome records.

Exclusion criteria: history of intracranial hemorrhage before admission; comorbid coagulation disorders or ongoing use of strong anticoagulants (e.g., warfarin with international normalized ratio >2.0, direct oral anticoagulants); admission platelet count <100 × 10^9^/L; severe liver or kidney dysfunction (e.g., estimated glomerular filtration rate [eGFR] <30 mL/min/1.73 m^2^); presence of malignant tumors, active gastrointestinal bleeding, or other bleeding tendencies; missing important clinical data that could not be assessed.

### 2.3. Grouping

In this study, patients were divided into 2 groups based on whether they received tirofiban treatment. To control for baseline confounding biases, PSM was used to perform 1:1 matching. Matching variables included age, admission NIHSS score, whether endovascular therapy was received, whether intravenous thrombolysis was performed, admission blood glucose levels, and eGFR. A caliper value of 0.02 was set for the matching process. A total of 50 matched pairs were successfully obtained, forming the case group (tirofiban treatment group) and the control group (nontirofiban treatment group), with 50 patients in each group. After matching, the baseline characteristics were balanced between the 2 groups. Although major baseline characteristics associated with stroke severity and bleeding risk were included in the propensity score model, some potentially relevant confounding factors, including prior antiplatelet therapy, history of oral diseases or periodontal conditions, and long-term glycemic variability indicators, were not incorporated into the matching process. Hemoglobin A1c (HbA1c) was analyzed separately in subgroup analysis because poor glycemic control was hypothesized to act not only as a confounder but also as a potential effect modifier influencing tirofiban-associated bleeding risk.

### 2.4. Data collection

Retrospective data collection was performed through the hospital’s electronic medical records system, stroke unit database, and imaging archiving system. All data were extracted by neurologists who had received standardized training and used a predesigned standardized case report form. To ensure data quality, another researcher, who was not involved in the grouping, rechecked 10% of randomly selected cases, with data consistency exceeding 95%.

#### 2.4.1. Baseline data collection

Baseline data for all included patients were collected, primarily including: demographic data: age, sex; stroke-related characteristics: NIHSS score at admission, time from symptom onset to hospital admission, stroke etiology classification; medical history: hypertension, history of stroke or transient ischemic attack, coronary heart disease history; and diabetes-related indicators: duration of diabetes, admission random blood glucose, and HbA1c levels.

#### 2.4.2. Treatment-related variables collection

Detailed records of the patients’ treatment plans were documented: tirofiban usage: whether tirofiban was used, timing of administration, intravenous bolus dose, continuous infusion dose, and total duration of treatment; reperfusion therapy: whether intravenous thrombolysis and/or endovascular therapy were performed; and other concomitant medications: usage of antiplatelet agents, antihypertensive medications, etc, before and after admission.

#### 2.4.3. Laboratory and imaging data collection

Key laboratory test results within 24 hours of admission were collected, including platelet count, international normalized ratio, and eGFR. In addition, the Alberta Stroke Program Early CT Score from the first noncontrast head computed tomography scan upon admission was recorded. Two neurologists, blinded to group allocation, independently assessed the computed tomography images. In cases of disagreement, consensus was reached through discussion.

#### 2.4.4. Outcome indicator data collection

The primary outcome of this study was overall extracranial bleeding events within 7 days after treatment, defined as any nonintracranial bleeding event that required clinical intervention or led to a hemoglobin decrease of ≥2 g/dL. These included gingival bleeding, epistaxis, subcutaneous hematomas, gastrointestinal bleeding, and urinary tract bleeding, among others. Intracranial hemorrhage events (both symptomatic and asymptomatic) were recorded separately as safety outcome measures. Secondary outcomes included: thrombocytopenia (defined as a platelet count <100 × 10^9^/L or a decrease >50% from baseline); neurological function and clinical outcomes: modified Rankin Scale (mRS) score at discharge (with a good outcome defined as mRS 0–2), NIHSS score at discharge, and all-cause mortality during hospitalization. All bleeding events were confirmed through a systematic review of medical records, nursing records, and laboratory test results.

### 2.5. Statistical analysis

Statistical analysis was performed using R software (version 4.3.0). Continuous variables were presented as mean ± standard deviation or median (interquartile range), with group comparisons conducted using *t* tests or Mann–Whitney *U* tests. Categorical variables were expressed as counts (%), and group comparisons were performed using the χ^2^ test or Fisher exact test. To control for confounding biases, PSM was used for 1:1 matching, with age, admission NIHSS score, endovascular treatment, intravenous thrombolysis, admission blood glucose, and eGFR as matching variables, and a caliper value of 0.02. Multivariable logistic regression analysis was used to identify independent factors associated with overall extracranial bleeding events, with results expressed as adjusted odds ratios (aOR) and 95% confidence intervals (CI). Subgroup analysis and interaction tests were conducted to explore effect differences among patients with different characteristics. All tests were two-sided, and a *P* value <.05 was considered statistically significant.

## 3. Results

### 3.1. Patient enrollment and baseline characteristics

After screening through the electronic medical record system and applying PSM, a total of 100 patients were included in the analysis, with 50 patients in the tirofiban group and 50 patients in the control group. As shown in Table 1, there were no statistically significant differences between the 2 groups in terms of demographic data, stroke severity and etiology, key treatment methods, medical history, diabetes-related indicators, or laboratory test results at admission (all *P* > .05). In addition, the standardized mean differences were all <0.1, indicating a good matching effect. The baseline characteristics were well-balanced between the 2 groups, ensuring comparability.

**Table 1:** Comparison of baseline characteristics after propensity score matching.

Baseline characteristic	Tirofiban group (n = 50)	Control group (n = 50)	*P* value	SMD
Demographics				
Age (yr), mean ± SD	65.8 ± 9.3	66.1 ± 8.7	.865	0.033
Male, n (%)	32 (64.0)	30 (60.0)	.687	0.082
Stroke severity, etiology, and treatment				
Admission NIHSS score, mean ± SD	12.5 ± 4.8	12.1 ± 5.0	.672	0.082
Onset-to-door time (h), median (IQR)	3.5 (2.0–6.0)	3.8 (2.2–6.5)	.721	0.065
TOAST classification, n (%)				0.091
Large-artery atherosclerosis	28 (56.0)	25 (50.0)		
Cardioembolism	15 (30.0)	14 (28.0)		
Other/undetermined etiology	7 (14.0)	11 (22.0)		
Underwent endovascular therapy, n (%)	35 (70.0)	36 (72.0)	.827	0.044
Underwent intravenous thrombolysis, n (%)	18 (36.0)	16 (32.0)	.673	0.084
Medical history and comorbidities				
History of hypertension, n (%)	38 (76.0)	40 (80.0)	.637	0.098
History of stroke/TIA, n (%)	10 (20.0)	8 (16.0)	.602	0.104
History of coronary artery disease, n (%)	12 (24.0)	10 (20.0)	.629	0.097
Diabetes-related metrics				
Diabetes duration (yr), median (IQR)	8.0 (4.0–14.0)	7.5 (3.0–13.0)	.589	0.112
Admission random glucose (mmol/L), mean ± SD	11.2 ± 3.5	10.9 ± 3.1	.634	0.091
HbA1c (%), mean ± SD	7.8 ± 1.5	7.6 ± 1.4	.487	0.137
Admission laboratory and imaging findings				
Admission systolic BP (mm Hg), mean ± SD	148 ± 22	145 ± 24	.521	0.129
Platelet count (×10^9^/L), mean ± SD	198 ± 51	205 ± 48	.472	0.142
eGFR (mL/min/1.73 m^2^), mean ± SD	85.6 ± 18.2	87.1 ± 17.5	.667	0.084
INR, mean ± SD	1.02 ± 0.08	1.01 ± 0.07	.495	0.133
Admission CT ASPECTS, median (IQR)	9 (8–10)	9 (8–10)	.845	0.039

ASPECTS = Alberta Stroke Program Early CT Score, BP = blood pressure, CT = computed tomography, eGFR = estimated glomerular filtration rate, HbA1c = glycated hemoglobin, INR = international normalized ratio, IQR = interquartile range, NIHSS = National Institutes of Health Stroke Scale, SD = standard deviation, SMD = standardized mean difference, TIA = transient ischemic attack, TOAST = Trial of Org 10172 in Acute Stroke Treatment.

### 3.2. Comparison of primary and secondary outcome events

The bleeding events and clinical outcomes in the 2 groups are compared in Table 2. The incidence of overall extracranial bleeding events (the primary outcome) was 32.0% (16/50) in the tirofiban group, significantly higher than the 16.0% (8/50) in the control group (risk ratio [RR] = 2.00, 95% CI = 1.11–3.60, *P* = .021). Among specific extracranial bleeding events, the incidence of gingival bleeding (10.0% vs 2.0%, *P* = .047) and thrombocytopenia (10.0% vs 2.0%, *P* = .047) was significantly higher in the tirofiban group. However, the incidence of other events such as subcutaneous bruising, epistaxis, and gastrointestinal bleeding showed no statistically significant differences between the 2 groups.

**Table 2:** Comparison of hemorrhagic events and clinical outcomes (n [%]).

Outcome measure	Tirofiban group (n = 50)	Control group (n = 50)	RR	95% CI	*P* value
Primary outcome: overall extracranial hemorrhagic events	16 (32.0)	8 (16.0)	2	1.11–3.60	.021
Types of extracranial hemorrhagic events					
Gingival bleeding	5 (10.0)	1 (2.0)	5	0.61–41.23	.047
Subcutaneous ecchymosis	4 (8.0)	2 (4.0)	2	0.38–10.49	.408
Epistaxis	3 (6.0)	1 (2.0)	3	0.32–27.89	.311
Gastrointestinal bleeding	2 (4.0)	2 (4.0)	1	0.14–6.98	1
Urinary tract bleeding	2 (4.0)	1 (2.0)	2	0.19–21.37	.564
Bleeding at other sites	0 (0)	1 (2.0)	–	–	.317
Intracranial hemorrhagic events (safety observation)					
sICH	0 (0)	0 (0)	–	–	–
Any intracranial hemorrhage	0 (0)	1 (2.0)	–	–	.317
Thrombocytopenia	5 (10.0)	1 (2.0)	5	0.61–41.23	.047

CI = confidence interval, RR = risk ratio, sICH = symptomatic intracranial hemorrhage.

Regarding intracranial hemorrhage, no sICH occurred in either group during the study period. One case of asymptomatic intracranial hemorrhage (2.0%) was observed in the control group, while no such events were observed in the tirofiban group, with no statistically significant difference between the groups (*P* = .317).

### 3.3. Multivariable logistic regression analysis of overall extracranial bleeding events

To further control for confounding factors and identify independent risk factors for overall extracranial bleeding events, we performed multivariable logistic regression analysis (Table 3). The results showed that after adjusting for factors such as age, stroke severity, endovascular treatment, blood pressure, and platelet count, tirofiban treatment (aOR = 2.45, 95% CI = 1.17–5.13, *P* = .018) and admission random blood glucose levels (aOR = 1.22, 95% CI = 1.02–1.46, *P* = .032) were independent risk factors for overall extracranial bleeding events. Age, admission NIHSS score, whether endovascular therapy was performed, admission systolic blood pressure, and platelet count were not independently associated with the risk of overall extracranial bleeding events.

**Table 3:** Multivariate logistic regression analysis for overall extracranial hemorrhagic events.

Variable	aOR	95% CI	*P* value
Tirofiban treatment (yes vs no)	2.45	1.17–5.13	.018
Age (per 1-yr increase)	1.01	0.96–1.06	.768
Admission NIHSS score (per 1-point increase)	1.06	0.96–1.16	.258
Admission random glucose (per 1 mmol/L increase)	1.22	1.02–1.46	.032
Underwent endovascular therapy (yes vs no)	1.42	0.61–3.31	.414
Admission systolic BP (per 10 mm Hg increase)	1.05	0.89–1.24	.546
Platelet count (per 50 × 10^9^/L increase)	0.88	0.66–1.17	.372

aOR = adjusted odds ratio, BP = blood pressure, CI= confidence interval, NIHSS = National Institutes of Health Stroke Scale.

### 3.4. Subgroup analysis of overall extracranial bleeding events

We performed a predefined subgroup analysis of overall extracranial bleeding events to explore the consistency of the risk associated with tirofiban treatment across different patient characteristics (Table 4). The interaction test showed that in most subgroups, tirofiban treatment was associated with a higher risk of extracranial bleeding, with consistent effect directions. However, a significant interaction was found in the blood glucose control subgroup (*P*-interaction = .04). Specifically, in patients with poor blood glucose control (HbA1c ≥7.5%), tirofiban treatment significantly increased the risk of extracranial bleeding (odds ratio [OR] = 3.33, 95% CI = 1.05–10.56). In contrast, in patients with good blood glucose control (HbA1c <7.5%), this increased risk trend did not reach statistical significance (OR = 1.33, 95% CI = 0.52–3.42). In other subgroups, such as whether endovascular therapy was performed, whether intravenous thrombolysis was administered, and admission stroke severity (NIHSS score), the interaction tests did not indicate significant effect modification (*P*-interaction >.05).

**Table 4:** Subgroup analysis for overall extracranial hemorrhagic events.

Subgroup	Total (N)	OR tirofiban vs control	95% CI	*P* for interaction
Overall	100	2	1.02–3.92	–
Underwent endovascular therapy				.65
Yes	71	1.85	0.87–3.93	
No	29	2.5	0.73–8.57	
Underwent intravenous thrombolysis				.42
Yes	34	3.12	0.78–12.52	
No	66	1.65	0.72–3.79	
Admission NIHSS score				.09
<12	48	1.2	0.44–3.30	
≥12	52	2.86	0.99–8.24	
HbA1c				.04
<7.5%	52	1.33	0.52–3.42	
≥7.5%	48	3.33	1.05–10.56	

CI = confidence interval, HbA1c = glycated hemoglobin, NIHSS = National Institutes of Health Stroke Scale, OR = odds ratio.

### 3.5. Other safety outcomes and neurological function prognosis

In addition to the primary focus on bleeding events, we analyzed other important safety and short-term prognosis indicators (Table 5). The results showed no significant differences between the 2 groups in terms of inpatient mortality (6.0% vs 4.0%, RR = 1.50, *P* = .648) and the incidence of nonintracranial hemorrhagic severe adverse events (such as severe infections, acute heart failure, etc; 10.0% vs 8.0%, RR = 1.25, *P* = .731). Regarding discharge destinations, most patients were discharged to home or rehabilitation facilities, with similar proportions between the 2 groups (*P* = .841).

**Table 5:** Other safety and prognostic outcomes.

Outcome measure	Tirofiban group (n = 50)	Control group (n = 50)	Statistics	*P* value
In-hospital mortality, n (%)	3 (6.0)	2 (4.0)	RR = 1.50 (0.26–8.64)	.648
Nonintracranial serious adverse events, n (%)	5 (10.0)	4 (8.0)	RR = 1.25 (0.35–4.42)	.731
Discharge disposition, n (%)				.841
Home/rehabilitation	40 (80.0)	39 (78.0)		
Other hospital/long-term care	10 (20.0)	11 (22.0)		
NIHSS improvement, median (IQR)	4 (2–7)	4 (1–6)	*Z* = −0.789	.43
Favorable outcome (mRS 0–2) at discharge, n (%)	18 (36.0)	16 (32.0)	RR = 1.13 (0.65–1.95)	.672

IQR = interquartile range, mRS = modified Rankin Scale, NIHSS = National Institutes of Health Stroke Scale, RR = risk ratio.

In terms of neurological outcomes, the proportion of patients with a good functional prognosis (mRS 0–2) at discharge was similar between the tirofiban group and the control group (36.0% vs 32.0%, RR = 1.13, *P* = .672). The median improvement in NIHSS scores from admission to discharge was 4 points in both groups, with no statistically significant difference between the groups (*P* = .43).

### 3.6. Sensitivity analysis

To assess the effectiveness of PSM and the impact of initial sample selection bias on the robustness of the primary outcome (overall extracranial bleeding events), we conducted a sensitivity analysis comparing the association between tirofiban and the risk of overall extracranial bleeding events before and after PSM (Table 6).

**Table 6:** Sensitivity analysis: association between tirofiban and overall extracranial hemorrhagic events before and after PSM.

Analysis cohort	Tirofiban group events/total (%)	Control group events/total (%)	RR	95% CI	*P* value
Before PSM (initial cohort)	30/85 (35.3)	22/110 (20.0)	1.77	1.10–2.85	.019
After PSM (matched cohort)	16/50 (32.0)	8/50 (16.0)	2	1.11–3.60	.021

CI = confidence interval, PSM = propensity score matching, RR = risk ratio.

Before matching, the incidence of overall extracranial bleeding events in the initial cohort was 35.3% (30/85) in the tirofiban group, significantly higher than the 20.0% (22/110) in the control group, with a crude risk ratio of 1.77 (*P* = .019). After balancing key baseline confounders through PSM, the RR increased slightly to 2.00 (*P* = .021), with a more precise CI. The results before and after matching both demonstrated a significant association between tirofiban treatment and an increased risk of overall extracranial bleeding events. This consistency indicates that our primary findings are robust to the matching process, minimizing bias caused by baseline imbalances.

## 4. Discussion

This study, through retrospective cohort analysis, explored the risk of overall bleeding events with tirofiban in patients with AIS and diabetes mellitus. The results indicated that tirofiban significantly increased the incidence of overall extracranial bleeding events (32.0% vs 16.0%, *P* = .021), with gingival bleeding and thrombocytopenia being the main manifestations. Notably, no sICH occurred in either group during the study period, indicating that the intracranial bleeding risk was very low in this rigorously selected population. This safety characteristic is consistent with recent studies that have reported the use of tirofiban under strict indications, suggesting that in AIS patients with diabetes, the primary safety concern with tirofiban may be extracranial bleeding and thrombocytopenia rather than fatal intracranial bleeding.^[10,11]^

From a pathophysiological perspective, the microvascular system in diabetic patients is chronically exposed to hyperglycemic conditions, which triggers a series of complex molecular cascades, including the accumulation of advanced glycation end products, increased oxidative stress, and abnormal activation of the protein kinase C pathway. These factors collectively lead to endothelial dysfunction and disruption of intercellular junctions.^[12,13]^ The oral gingival mucosa, as a “window” to the systemic endothelial function, has rich blood supply, dense capillary networks, and thick epithelium, making it particularly sensitive to metabolic abnormalities and drug effects.^[14]^ When potent antiplatelet agents like tirofiban are introduced, their powerful inhibition of platelet aggregation through blocking the platelet glycoprotein IIb/IIIa receptors could induce a bleeding tendency in the already dysfunctional endothelial system of the extracranial microvascular bed (especially the oral mucosa), triggered by spontaneous or minor trauma. This provides a plausible mechanistic explanation for the significantly higher incidence of gingival bleeding observed.^[15]^ In addition, immune-mediated platelet destruction or dysfunction induced by tirofiban may further synergistically increase the bleeding risk.^[16]^

This study also found that elevated admission random blood glucose levels were an independent risk factor for overall extracranial bleeding events (aOR = 1.22, *P* = .032), which is consistent with the pathophysiological basis of hyperglycemia directly impairing vascular endothelial integrity, inhibiting platelet function, and prolonging bleeding time.^[17,18]^ Importantly, subgroup analysis revealed that in patients with poor blood glucose control (HbA1c ≥7.5%), the risk of extracranial bleeding associated with tirofiban was further significantly increased (OR = 3.33, *P*-interaction = .04). This strongly suggests that chronic poor blood glucose control and acute-phase antiplatelet therapy have an adverse synergistic effect, significantly amplifying the bleeding risk, and blood glucose control levels are a key modifiable factor in regulating the safety of tirofiban.^[19]^ The innovation of this study mainly lies in its focus on a specific high-risk subgroup of AIS patients with diabetes mellitus and its comprehensive evaluation of extracranial bleeding events, rather than in a novel intervention strategy itself. Therefore, the conclusions of the present study should be interpreted cautiously and are mainly applicable to diabetic AIS patients treated in similar real-world clinical settings. Extrapolation of these findings to the general AIS population or to patients with substantially different baseline bleeding profiles requires further validation through large-scale prospective multicenter studies.

Several limitations of this study should be acknowledged. First, this was a single-center retrospective study with a relatively small sample size, which may limit the external validity and generalizability of the findings. Second, residual confounding could not be completely eliminated because of the retrospective design, particularly regarding unmeasured variables such as previous antiplatelet exposure, oral mucosal conditions, and physician-dependent treatment selection. Third, most extracranial bleeding events observed in this study were mild-to-moderate in severity, and their long-term impact on functionalrecovery, treatment adherence, and quality of life was not evaluated. Fourth, this study did not further assess safety differences according to tirofiban dosage, administration timing, infusion duration, or different concomitant antithrombotic regimens. Finally, the relatively short follow-up period may have underestimated delayed bleeding complications and long-term neurological outcomes.

The clinical significance of this study lies in its systematic shift of safety monitoring focus for tirofiban in AIS patients with diabetes from the traditional concern of intracranial hemorrhage to the more common but often overlooked extracranial bleeding events (especially gingival bleeding) and thrombocytopenia. For such patients, routine evaluation of blood glucose control (HbA1c) and oral examination should be performed before administering tirofiban. In patients with poor blood glucose control, a more cautious approach should be taken to weigh the clinical benefits of revascularization or antithrombotic therapy against the potential bleeding risks, and oral hygiene education should be enhanced during medication use. Patients should use a soft-bristled toothbrush, regularly check for gingival bleeding tendencies, and closely monitor dynamic changes in platelet count. Another important clinical implication of the present study is the potential “risk–reward imbalance” associated with tirofiban use in diabetic AIS patients. Although extracranial bleeding events and thrombocytopenia were significantly increased, no corresponding improvement in short-term neurological outcomes or functional prognosis was observed. This finding suggests that intensified antiplatelet therapy may expose certain diabetic patients to additional safety risks without providing substantial neurological benefit. In addition, the absence of sICH in our cohort should be interpreted cautiously. The relatively limited sample size, retrospective design, and strict patient selection criteria may have contributed to an underestimation of the true intracranial bleeding risk associated with tirofiban.

Furthermore, the results of this study reiterate the multiple benefits of intensifying blood glucose control during both acute and long-term stroke management to reduce treatment-related complications and improve overall patient prognosis. Future studies could further explore the effectiveness of preventive oral care measures, risk stratification based on admission blood glucose or HbA1c levels, individualized dosing regimens, and the safety comparison between tirofiban and other antiplatelet drugs, with the aim of developing optimized and safer antiplatelet treatment strategies for this high-risk population.

## Author contributions

**Conceptualization:** Dongmei Rao, Zhi Li, Biao Guo.

**Data curation:** Dongmei Rao, Zhi Li, Biao Guo.

**Formal analysis:** Dongmei Rao, Zhi Li, Biao Guo.

**Funding acquisition:** Dongmei Rao.

**Writing – original draft:** Dongmei Rao.

**Writing – review & editing:** Dongmei Rao.
